# Supplementary material for: Effect of Soil Acidification on the Production of Se-Rich Tea
Source: Plants (Basel). 2023 Aug 7;12(15):2882. doi: 10.3390/plants12152882 (PMC10420883; doi:10.3390/plants12152882)
Supplement: Supplementary file 1 [file plants-12-02882-s001.zip › plants-2477998-supplementary.pdf]

Table S1. Coordinates of the study area.

No.	Abbreviation	Coordinates
1	YJW	31°16′33″N 119°0′54″E
2	YX	31°8′6″N 119°26′54″E
3	SD	31°15′8″N 119°35′26″E
4	FR	31°10′46″N 119°26′15″E
5	GM	31°33′19″N 119°9′31″E
6	CKS	31°17′52″N 119°28′55″E
7	LX	31°12′29″N 119°39′20″E
8	LG	31°13′58″N 119°45′48″E

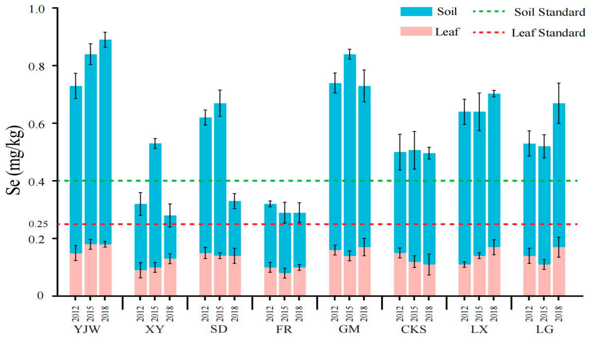

Figure S1 The content of selenium in the soil of the sampling sites and the content of Se in shoots of tea trees were studied in tea plantations.

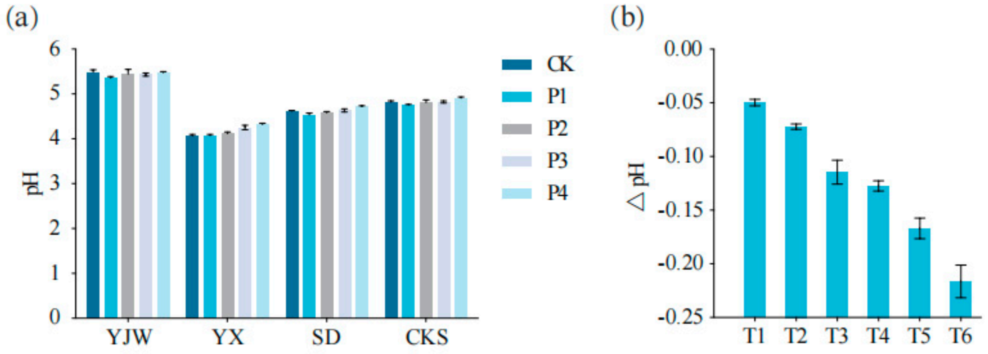

Fig. S2 The change of pH content for different treatments：(a) pH changes at four sampling sites in simulated acid rain experiments; (b) pH changes under different acidification treatments.
